# Velpeau’s Operative Surgery

**Published:** 1840-10

**Authors:** 


VELPEAU'S OPERATIVE SURGERY.

A translation of this work is now in progress by John R. W. Dunbar, M. D., Professor of Surgery in the Washington Medical College, Baltimore, and will be published in the course of next spring. Velpeau's Surgery is one of the ablest works on that subect extant in any language. It will be accompanied by notes on American Surgery by the Translator, and executed with the ability of which the character of Professor Dunbar gives promise, will no doubt prove highly acceptable to the American profession. It should be mentioned, that this is the second edition of Velpeau's Surgery, much enlarged and improved. Y.

